# The role of the *CEBPB* gene in porcine adipogenesis: a study using CRISPR/Cas9-edited mesenchymal stem cells

**DOI:** 10.1007/s13353-025-01030-x

**Published:** 2025-11-26

**Authors:** Mehmet Onur Aksoy, Jedrzej Rozynek, Monika Stachowiak, Izabela Szczerbal

**Affiliations:** Department of Genetics and Animal Breeding, Poznan University of Life Sciences, Wolynska 33, Poznan, 60-637 Poland

**Keywords:** Adipogenesis, CEBPB, CRISPR/Cas9, MSC, Pig, Proliferation

## Abstract

Clustered Regularly Interspaced Short Palindromic Repeats (CRISPR)/Cas9 is a powerful tool for gene editing and the regulation of gene expression. It enables the introduction of targeted mutations, thereby facilitating functional studies of specific genes in various cellular processes. In this study, we aimed to generate a deletion in the promoter region of the *CEBPB* gene, which encodes a transcription factor involved in adipogenesis, and to evaluate the impact of this modification on the adipogenic differentiation potential of porcine mesenchymal stem cells (MSCs). A 575-bp deletion was introduced in the target region, resulting in the generation of both homozygous and heterozygous mutant cells. Adipogenic differentiation was assessed by quantifying transcript levels of adipocyte marker genes (*GATA2*, *CEBPA*, *PPARG*, and *FABP4*) at days 0, 4, 6, 8, and 10 of the differentiation process. Disruption of *CEBPB* expression led to the downregulation of these adipogenic markers, indicating impaired adipocyte differentiation. Additionally, to assess the proliferative capacity of the edited cells, the expression levels of proliferation-associated genes (*CCND1*, *MCM2*, and *PCNA*) were measured. A reduction in their transcript levels was observed in both homozygous and heterozygous mutant cells. These findings indicate that both homozygous and heterozygous deletions in the *CEBPB* promoter completely block adipogenesis and alter MSC proliferation, highlighting the pivotal role of *CEBPB* not only in adipogenic differentiation but also in the regulation of cell proliferation in porcine mesenchymal stem cells. These results provide new insights into the molecular mechanisms underlying adipose tissue development and have implications for pig breeding strategies aimed at optimizing carcass composition, as well as for biomedical research focused on adipose tissue biology.

**Supplementary Information:**

The online version contains supplementary material available at 10.1007/s13353-025-01030-x.

## Introduction

Adipogenesis—the process of fat cell development—is of particular interest in pig research, as adipose tissue significantly influences growth rates, energy balance, and survival during early life, as well as traits relevant to meat quality and agricultural productivity. Furthermore, pigs are widely recognized as valuable biomedical models for human diseases, including obesity (Louveau et al. 2016; Stachowiak et al. 2016). Adipogenesis is regulated by a complex network of transcription factors that either promote or inhibit adipocyte differentiation. Among these, peroxisome proliferator-activated receptor gamma (PPARγ) and members of the CCAAT/enhancer-binding protein (C/EBP) family play central roles in orchestrating this process (Rosen and MacDougald 2006; Farmer 2006).

One of the transcription factors in the C/EBP family is CCAAT/enhancer-binding protein beta (C/EBPβ), which contains a basic leucine zipper (bZIP) domain and acts as an early regulator of adipogenesis (Guo et al. 2015). C/EBPβ activates the expression of two master regulators, C/EBPα and PPARγ, which in turn drive the expression of a wide array of adipocyte-specific genes responsible for lipid metabolism, insulin sensitivity, and other adipocyte functions. The *CEBPB* gene, which encodes C/EBPβ, is intron-free and broadly expressed across various cell types, where it contributes to cell differentiation, proliferation, and the regulation of immune and inflammatory responses (Pal et al. 2009; Ren et al. 2023).

The role of C/EBPβ in adipogenesis has been extensively studied in rodent and human models. For example, RNA interference-mediated knockdown of *Cebpb* in murine 3T3-L1 cells inhibits adipogenesis, and *Cebpb* knockout mice exhibit reduced fat accumulation (Zhang et al. 2011; Tanaka et al. 1997). Conversely, overexpression of C/EBPβ induces adipocyte differentiation in 3T3-L1 cells even in the absence of hormonal inducers (Yeh et al. 1995). C/EBPβ has also been shown to be essential for mitotic clonal expansion (MCE), a phase during which growth-arrested preadipocytes re-enter the cell cycle and undergo several rounds of division prior to differentiation (Tang et al. 2003). Although MCE appears to be required for murine adipogenesis, some studies suggest it may not be essential (Qiu et al. 2001), and it is reportedly dispensable in human adipocytes (Marquez et al. 2017).

Despite its well-documented role in other species, the regulatory function of C/EBPβ during porcine adipogenesis remains poorly understood. In an in vitro model using mesenchymal stem cells (MSCs) derived from porcine bone marrow, *CEBPB* expression was shown to be upregulated during the early stages of adipogenic differentiation and downregulated at later stages (Szczerbal et al. 2009). Similarly, studies in adipose-derived MSCs (AD-MSCs) demonstrated that *CEBPB* exhibits a peak of transcriptional activity on day 4 of differentiation (Aksoy et al. 2024). Interestingly, *CEBPB* was the only gene upregulated in AD-MSCs with a disrupted *SREBF1c* gene, suggesting a functional interaction between *CEBPB* and *SREBF1c* (Aksoy et al. 2024). Reciprocal regulation between C/EBPβ and SREBP-1c has also been demonstrated, with each capable of activating the other’s promoter (Le Lay et al. 2002; Payne et al. 2009). Other studies have shown that the downregulation of the C/EBP family, including *CEBPB*, in porcine intramuscular preadipocytes leads to decreased *PPARG* expression, and the interaction between *CEBPB* and *FGF21* appears to play a significant role in adipogenesis (Wang et al. 2015). Furthermore, in a recent transcriptomic study comparing synovial MSCs from two pig breeds differing in growth and fat deposition, *CEBPB* was among the genes upregulated during the early stages of differentiation, when cells are actively proliferating (Ponsuksili et al. 2024).

Recent advances in CRISPR-based technologies have revolutionized the precision engineering of various genomic elements, including both coding and regulatory sequences. The ability to introduce targeted promoter deletions and to monitor cellular responses during key biological processes, such as adipogenesis, provides a powerful framework for elucidating gene function, which is of major importance for both biomedical and agricultural applications (Li et al. 2023; Fu et al. 2024). Novel approaches such as CRISPR interference (CRISPRi) and CRISPR activation (CRISPRa), which employ catalytically inactive Cas9 (dCas9) to repress or activate gene expression, open new directions in stem cell engineering (Razavi et al. 2024a). Furthermore, the integration of CRISPR systems with microfluidic-based biosensors has enabled the creation of robust platforms for highly sensitive early diagnosis, precise disease monitoring, and real-time genetic analysis (Razavi et al. 2024b, c).

In the present study, we aimed to further elucidate the transcriptional regulatory role of *CEBPB* during porcine adipogenesis. We employed the CRISPR/Cas9 genome editing system to generate targeted deletions in the promoter region of the *CEBPB* gene in porcine MSCs. The creation of both homozygous and heterozygous knockout variants enabled us to investigate how *CEBPB* disruption affects the proliferation and differentiation potential of MSCs into adipocytes, thereby providing new insights into its functional role in porcine adipose tissue development.

## Material and methods

### Cell culture and induction of adipogenesis

Adipose tissue-derived mesenchymal stem cells (MSCs) were cultured in Advanced DMEM medium (Gibco) supplemented with 10% fetal bovine serum (FBS; Sigma-Aldrich), 5 ng/mL fibroblast growth factor-2 (FGF-2; PromoKine), 2 mM L-glutamine (Gibco), 1 mM 2-mercaptoethanol (Sigma-Aldrich), 1× antibiotic-antimycotic solution (Sigma-Aldrich), and 1× MEM non-essential amino acids (NEAA; Gibco). Cells were maintained at 37 °C in a humidified atmosphere containing 5% CO₂. Upon reaching confluence, adipogenic differentiation was induced by replacing the growth medium with differentiation medium composed of Advanced DMEM (Gibco), 10% FBS (Sigma-Aldrich), 1× antibiotic-antimycotic solution (Sigma-Aldrich), 5 ng/mL FGF-2 (PromoKine), 1× linoleic acid–albumin solution (Sigma-Aldrich), 1× insulin–transferrin–selenium (ITS) supplement (Sigma-Aldrich), 1 µM dexamethasone (Sigma-Aldrich), 100 µM indomethacin (Sigma-Aldrich), and 50 µM isobutylmethylxanthine (IBMX; Sigma-Aldrich). Differentiation was carried out over a period of 10 days, with the medium replenished daily. The accumulation of lipid droplets was monitored by visual examination under phase-contrast microscopy (TS100 Eclipse, Nikon, Melville, NY, USA) and BODIPY staining according to procedure described by Aksoy et al. (2024).

### gRNA design and MSC transfection

Guide RNAs (gRNAs) targeting the promoter region upstream of the *CEBPB* gene were designed using the CRISPOR tool (http://crispor.tefor.net/). The gRNA sequences are listed in Table S1. Each gRNA oligonucleotide pair was cloned separately into the pX330 SpCas9 with PuroR vector (based on Addgene plasmid #4223, kindly provided by Dr. T. Flisikowska from Chair of Livestock Biotechnology, TUM, Freising, Germany), which contains a U6 promoter to drive gRNA expression, an sgRNA scaffold, and a puromycin resistance gene for selection. A total of 2 µg of plasmid DNA, derived from two gRNA-containing vectors, was co-transfected into MSCs using the P2 Primary Cell 4D-Nucleofector™ X Kit (Lonza) and the 4D-Nucleofector™ System (Lonza). To assess transfection efficiency, a control plasmid encoding GFP (pmaxGFP Vector; Lonza) was used. Following transfection, cells were seeded in six-well plates and incubated for 24 h. Puromycin selection (2 µg/mL) was then applied for 48 h to enrich for transfected cells. After selection, surviving cells were cultured further to allow for the formation of single-cell colonies.

### PCR genotyping to detect deletions in the 5′ regulatory region of *CEBPB*

To verify the introduction of deletions in the 5′ regulatory sequence of the *CEBPB* gene, PCR-based genotyping was performed on single-cell-derived MSC colonies. Genomic DNA was extracted from both transfected and wild-type (WT) MSCs using the MasterPure Complete DNA & RNA Purification Kit (Biosearch Technologies), according to the manufacturer’s instructions. Primers for genotyping were designed using Primer3Plus software (sequences provided in Table S2). PCR amplification of the wild-type allele yielded a product of 818 base pairs (bp), whereas a 243-bp shorter amplicon was observed in colonies carrying the deletion within the 5′ regulatory region of *CEBPB*.

### Sanger DNA sequencing

PCR products were purified using Exonuclease I and FastAP Thermosensitive Alkaline Phosphatase (Thermo Fisher Scientific), following the manufacturer’s protocol. Sequencing reactions were carried out using the BigDye Terminator v3.1 Cycle Sequencing Kit (Thermo Fisher Scientific). The resulting sequencing products were purified using Sephadex G-50 columns (Sigma-Aldrich) prior to capillary electrophoresis on a 3130 Genetic Analyzer (Applied Biosystems). The resulting chromatograms were analyzed using the SeqMan Pro software (DNASTAR) and aligned against the porcine *CEBPB* reference sequence (ENSSSCG00000034207, https://www.ensembl.org/).

### RNA isolation, cDNA synthesis, and real-time PCR

Total RNA was extracted from MSCs using TriPure Isolation Reagent (Roche), following the manufacturer’s instructions. RNA concentration and purity were assessed using a NanoDrop 2000 spectrophotometer (Thermo Scientific). One microgram of total RNA was reverse transcribed into cDNA using the Transcriptor First Strand cDNA Synthesis Kit (Roche). Real-time PCR primers were designed to span exon-exon junctions based on the Sscrofa11.1 reference genome (primer sequences are listed in Table S3). Quantitative PCR reactions were performed in triplicate using the LightCycler 480 II system (Roche) and the LightCycler 480 SYBR Green I Master Kit (Roche). Relative mRNA levels were calculated using the second derivative maximum method (Roche). The *RPL27* gene was used as the internal reference, and normalization was performed using the mean relative expression values of biological replicates.

### Statistical analysis

The statistical analysis was conducted using IBM SPSS Statistics 28 software, with a significance level set at 0.05. To examine changes between two groups over consecutive days, a repeated measures MANOVA test was employed. Additionally, Pillai’s trace and partial eta squared were calculated to evaluate the study’s effect sizes. To assess differences in quantitative data between groups, a non-parametric test was applied due to the very small number of measurements. Specifically, the Wilcoxon test with the Holm-Bonferroni correction was used, as it is recommended for extremely small groups.

## Results

### Characterization of the 5′ regulatory region of the porcine *CEBPB* gene

**Fig. 1:**
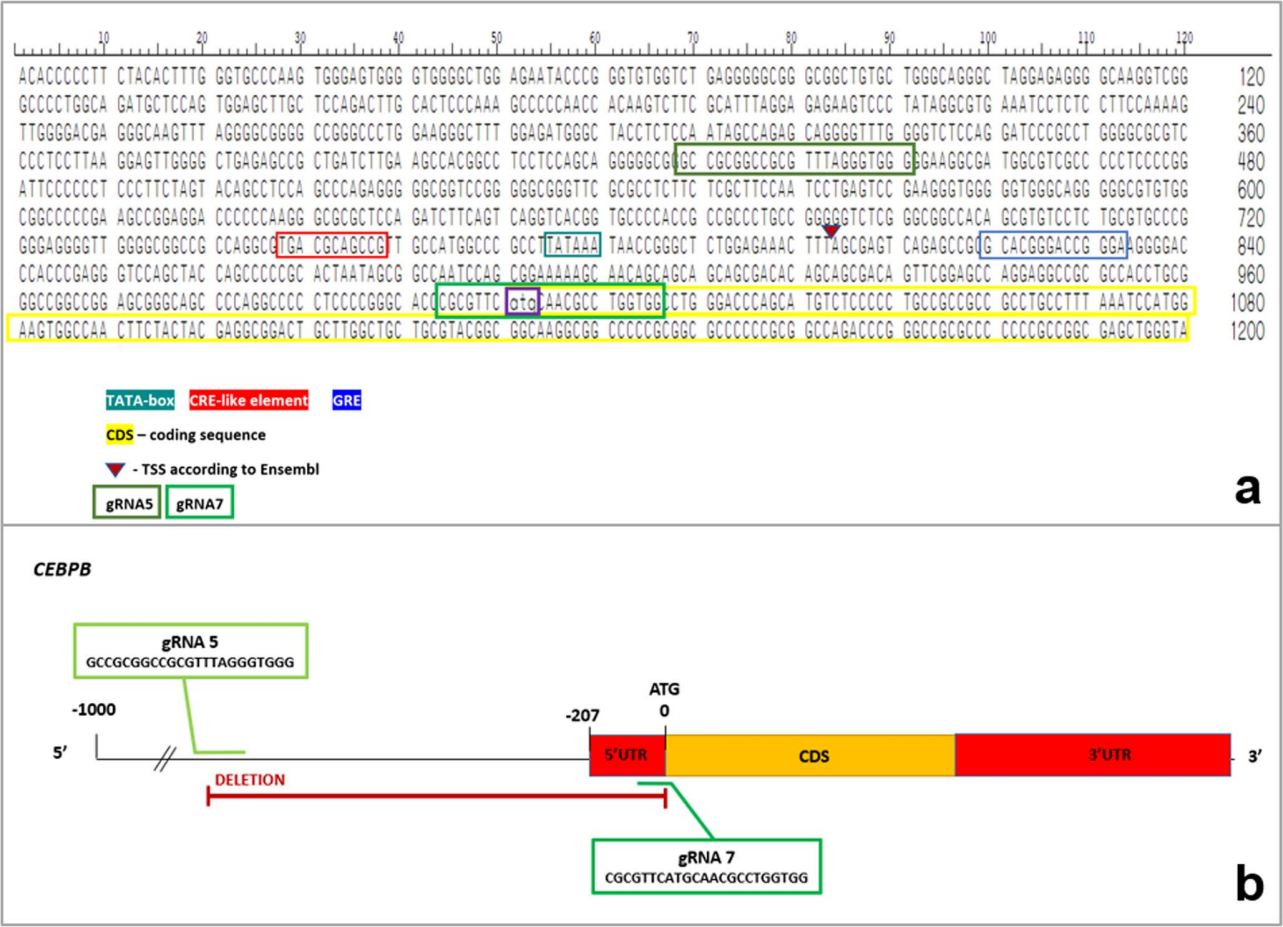
Schematic representation of the 5′ regulatory region of the *CEBPB* gene showing predicted transcription factor binding sites relevant to adipogenesis, including TATA box, CRE-like elements, GRE, CDS, and TSS. The positions of gRNA5 and gRNA7 used to delete the promoter region are indicated (**a**). Genomic structure of the porcine *CEBPB* gene with the locations of the designed gRNAs used for CRISPR/Cas9-mediated editing (**b**)

An in silico analysis of the 5′ regulatory sequence of the porcine *CEBPB* gene was conducted to identify putative promoter elements and transcription factor binding sites associated with adipogenesis. The analysis was performed using the DNASTAR Lasergene software suite (https://www.dnastar.com/software/). Multiple regulatory motifs were identified, including a TATA box, CRE-like elements, glucocorticoid response elements (GRE), a coding sequence start (CDS), and the transcription start site (TSS) region (Fig. 1a). To excise this regulatory element-rich region, two out of four designed single guide RNAs (gRNAs) were selected for CRISPR/Cas9 targeting, resulting in the generation of two cleavage sites flanking the region of interest (Fig. 1b; Table S1).

### Introduction of a deletion in the *CEBPB* locus

**Fig. 2:**
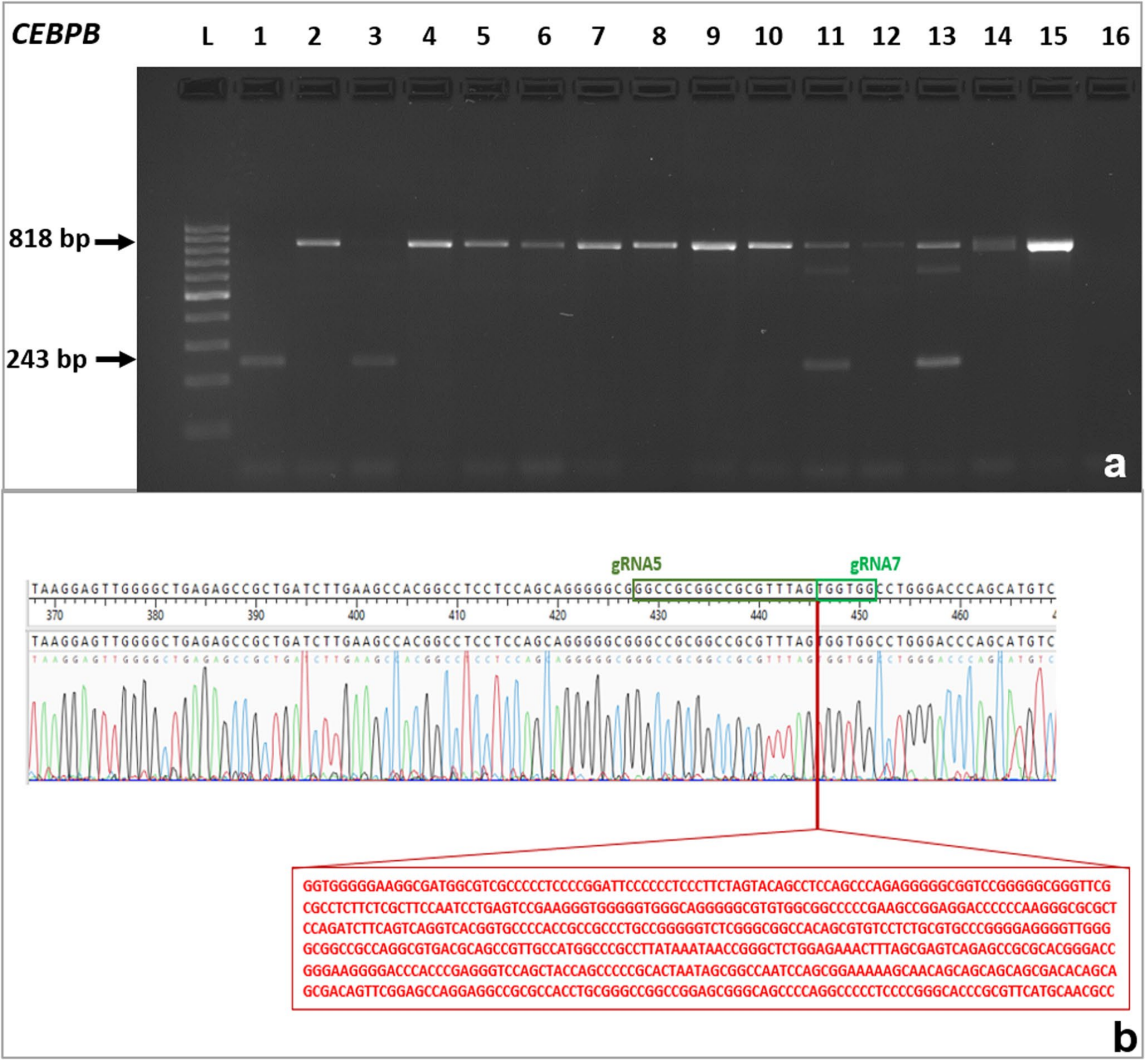
Representative electrophoresis image of PCR genotyping from single-cell-derived colonies, showing detection of the *CEBPB* promoter deletion. L – 100 bp DNA ladder (Thermo Fisher Scientific); Lanes 2, 4–10, 12, 14 – wild-type colony (818 bp); lane 1 – colony with a homozygous deletion (243 bp); lanes 3,11,13 – colonies with a heterozygous deletion; lane 15 - negative control (non-transfected cells); lane 16 - negative control (no DNA template) (a). Sanger sequencing chromatogram confirming a 575 bp deletion in the 5′ regulatory region of the *CEBPB* gene. The sequence shows the junction between the excised DNA ends, with partial gRNA5 and gRNA7 recognition sequences indicated (b)

CRISPR/Cas9-mediated genome editing was employed to introduce a targeted deletion within the *CEBPB* locus. Guide RNAs were cloned into the pX330 plasmid vector, and MSCs were transfected via nucleofection. Transfection efficiency was determined using the pmaxGFP control plasmid (Lonza, Basel, Switzerland) by counting GFP-positive MSCs in randomly selected microscopic fields. The mean efficiency was estimated at approximately 60% (Fig. S1). Following transfection, single MSC colonies were genotyped by PCR. Primers were designed to amplify genomic DNA regions flanking the gRNA target sites. In wild-type cells, the expected PCR product was 818 base pairs (bp), whereas cells carrying the intended deletion yielded a 243-bp fragment, indicating a 575-bp deletion in the *CEBPB* promoter region (Fig. 2a). Based on this genotyping approach, a total of eleven clones with targeted deletions were identified—one homozygous and ten heterozygous. The editing efficiency at the *CEBPB* locus reached 22.9%, as determined by the proportion of edited clones (*n* = 11) among all screened samples (*n* = 48). Sanger sequencing of PCR amplicons confirmed the presence of a 575-bp deletion within the 5′ regulatory region of the *CEBPB* gene (Fig. 2b). No additional mutations, insertions or deletions (indels), or partial deletions were detected apart from the intended 575-bp deletion in the target region.

### Expression profile of the *CEBPB* gene in wide-type MSC (MSC_WT_) and in MSC with deletion in promoter region (MSC_DEL_)

**Fig. 3:**
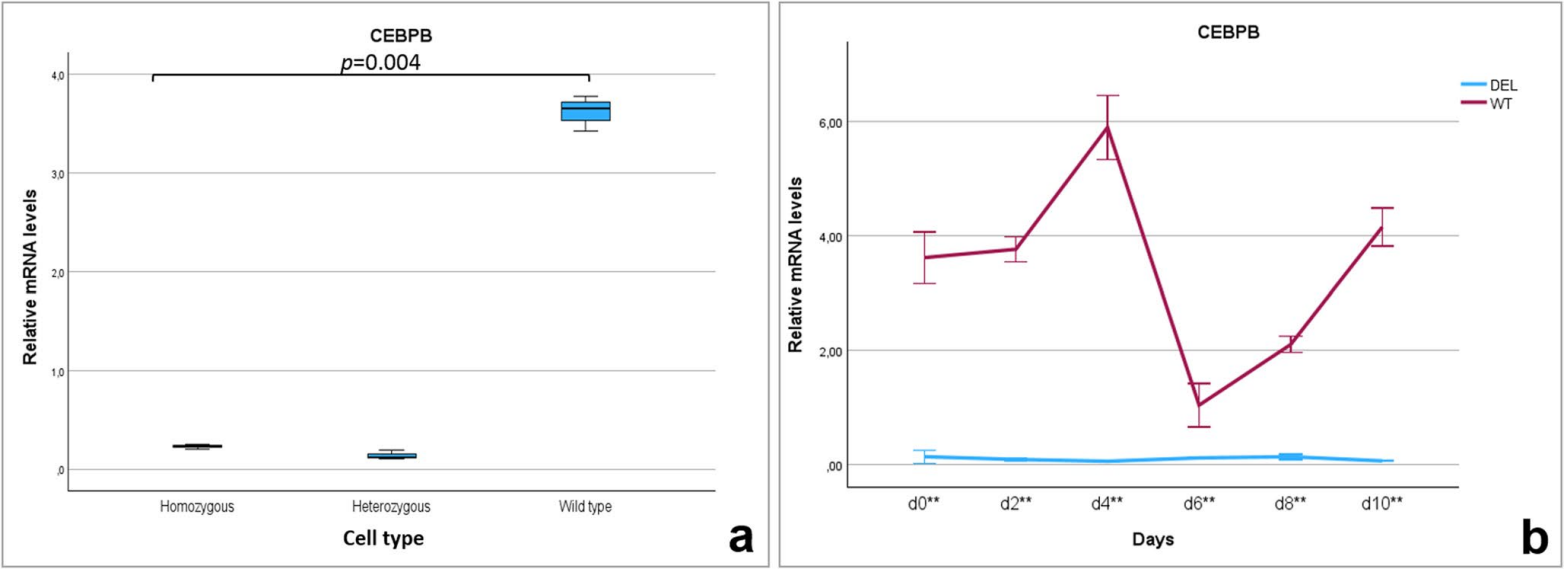
Relative transcript levels of *CEBPB* gene in wide-type MSC (MSC_WT_) and in MSC with deletion in promoter region (MSC_DEL_) in homozygous and heterozygous cells, Wilcoxon test, *p* < 0.004 (a) and during subsequent days of adipogenesis for heterozygous cells (b). The error bars represent 95% confidence intervals. **: significantly higher in MSC_WT_ than in MSC_DEL_, MANOVA test, *p* < 0.001

To assess whether the deletion introduced in the promoter region of the *CEBPB* gene affected its transcriptional activity, relative mRNA expression levels were compared between wild-type cells (MSC_WT_) and genetically modified cells (MSC_DEL_) carrying either homozygous or heterozygous deletions. A significant overall difference in *CEBPB* transcript levels was observed among cell types (Wilcoxon signed-rank test, *p* = 0.004). Post hoc analysis revealed that MSC_WT_ exhibited significantly higher *CEBPB* expression than MSC_DEL_ with heterozygous deletions (*p* = 0.025) and homozygous deletions (*p* = 0.025) (Fig. 3a). *CEBPB* expression in wild-type cells was approximately 11-fold higher than in heterozygous cells. Due to the markedly reduced proliferation of MSCs carrying a homozygous deletion, these cells could not be used for adipogenic differentiation analysis. Therefore, further differentiation studies were conducted only on MSC_DEL_ cells with heterozygous deletions, in comparison to MSC_WT_. The expression profile of *CEBPB* was evaluated over the course of adipogenic differentiation, from day 0 (undifferentiated MSCs) to day 10. In MSC_WT_, *CEBPB* exhibited a characteristic peak in transcript levels on day 4 of differentiation. In contrast, MSC_DEL_ cells showed significantly reduced *CEBPB* expression across all examined time points (MANOVA, *p* < 0.001) (Fig. 3b). These results demonstrate that the targeted promoter deletion effectively disrupted *CEBPB* expression, confirming the functional importance of this regulatory region during adipogenic differentiation.

### Expression patterns of adipogenesis-related genes in MSC_WT_ and MSC_DEL_

**Fig. 4:**
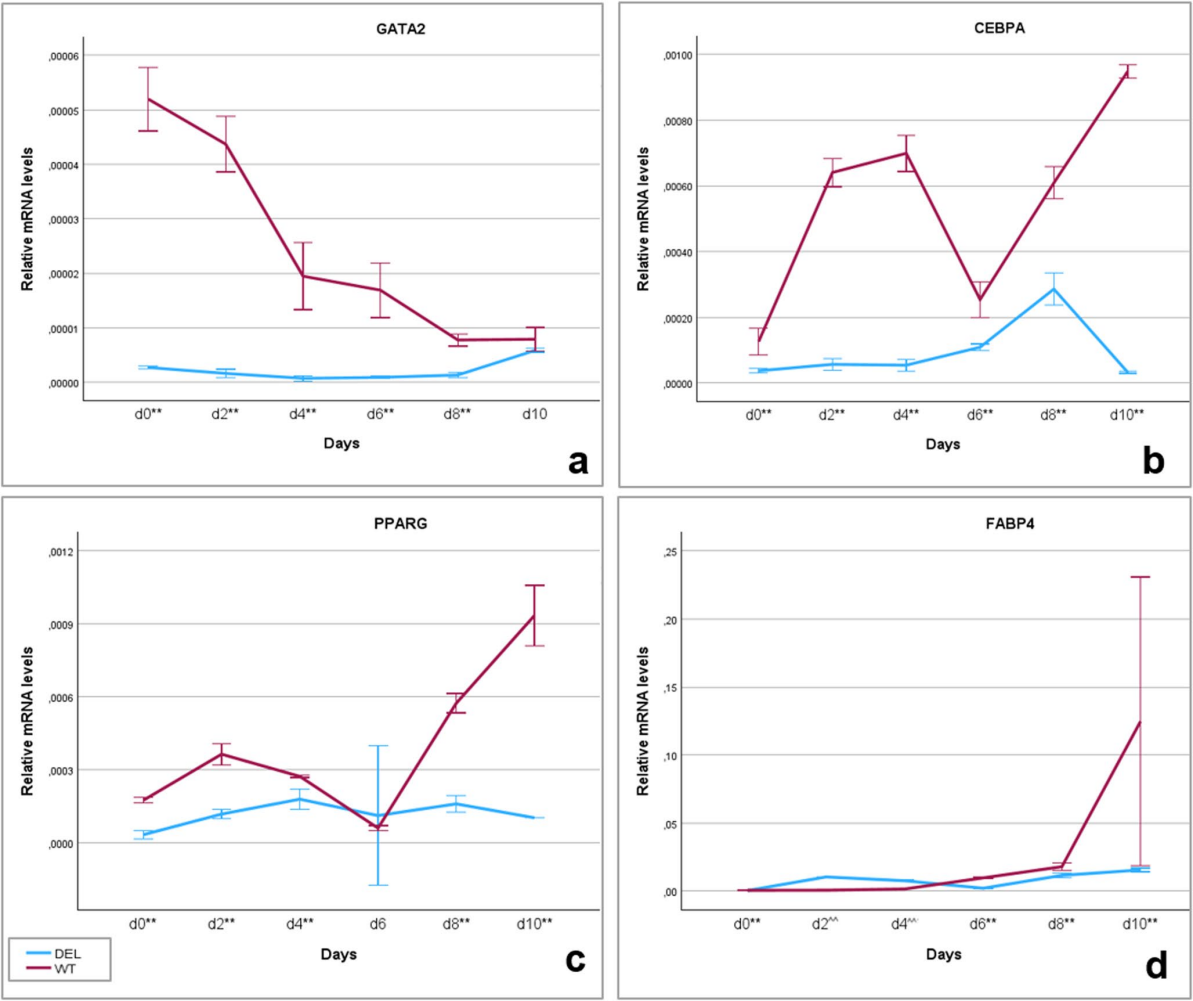
Relative transcript levels of *GATA2* (**a**), *CEBPA* (**b**), *PPARG* (**c**) and *FABP4* (**d**) during subsequent days of adipogenesis in MSC_WT_ and MSC_DEL_. Error bars represent 95% confidence intervals. **: significantly higher in MSC_WT_ than in MSC_DEL_, *p* < 0.001; ^^: significantly lower in MSC_WT_ than in MSC_DEL_, MANOVA test, *p* < 0.001

To assess the functional role of *CEBPB* in porcine adipogenesis, the expression profiles of key adipogenic marker genes were analyzed over the course of differentiation in MSC_WT_ and MSC_DEL_ (heterozygous) cells. In MSC_WT_, the highest expression level of *GATA2* was observed at day 0, followed by a gradual decrease throughout the differentiation process. In contrast, MSC_DEL_exhibited significantly lower *GATA2* expression compared to MSC_WT_ at all time points except day 10 (MANOVA, *p* < 0.001) (Fig. 4a). *CEBPA* expression in MSC_WT_ progressively increased during adipogenesis, reaching its peak at day 10. In * MSC*_DEL_, *CEBPA* transcript levels were significantly lower than those in MSC_WT_ across all days of differentiation (MANOVA, *p* < 0.001) (Fig. 4b). A significant induction of *PPARG* expression was detected in MSC_WT_ starting from day 6. In MSC_DEL_, *PPARG* expression was significantly reduced relative to MSC_WT_ at all time points, except on day 6 (MANOVA, *p* < 0.001) (Fig. 4c). The expression of *FABP4*, a marker of mature adipocytes, was also altered. MSC_DEL_ exhibited significantly lower *FABP4* transcript levels than MSC_WT_ at days 0, 6, 8, and 10, while significantly higher levels were observed at days 2 and 4 (MANOVA, *p* < 0.001) (Fig. 4d).

These results demonstrate that disruption of *CEBPB* expression interferes with the normal transcriptional cascade of adipogenesis, suppressing the expression of upstream transcription factors (*GATA2*,* CEBPA*,* PPARG*) as well as *FABP4*, a gene characteristic of terminal adipocyte differentiation. The downregulation of these markers corresponded with the observed phenotypic effect—cells with the *CEBPB* promoter deletion failed to accumulate lipid droplets, indicating an absence of adipogenic differentiation at the cellular level (Fig. S2).

### Assessment of the proliferative status of MSC_WT_ and MSC_DEL_

**Fig. 5:**
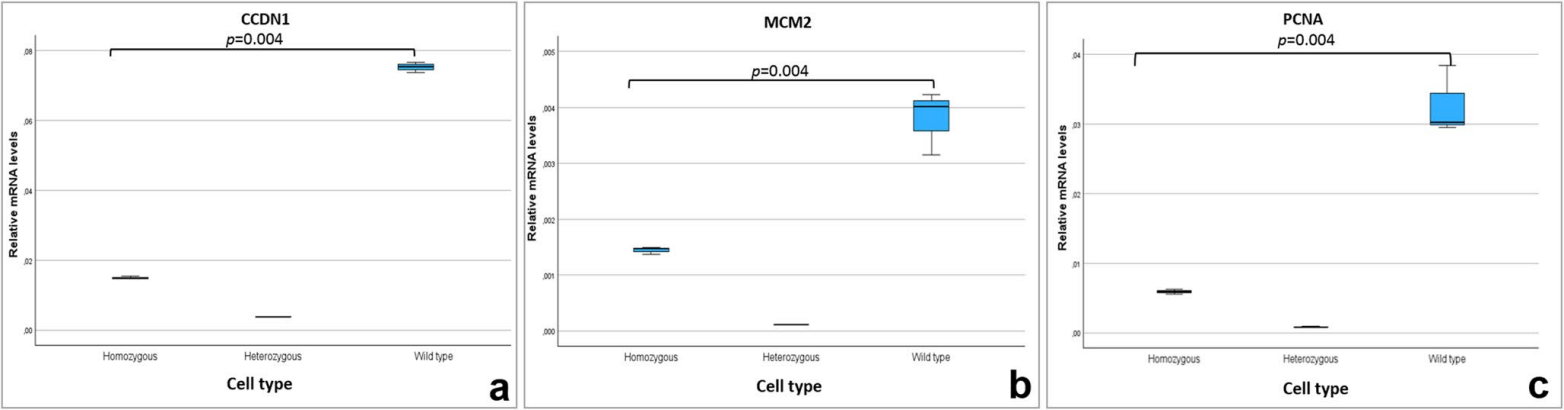
Relative transcript levels of *CCDN1* (a), *MCM2* (b) and *PCNA* (c) genes in wide-type MSC (MSC_WT_) and in MSC with deletion in promoter region of *CEBPB* gene (MSC_DEL_) in homozygous and heterozygous status, Wilcoxon test, *p* < 0.004

Due to the observed reduction in proliferation in *CEBPB*-modified cells, additional analyses were conducted to assess the expression of three proliferation-associated marker genes: *CCND1*, *MCM2*, and *PCNA*. A significant decrease in transcript levels of all three genes was detected in MSC_DEL_ cells —both heterozygous and homozygous—compared to unmodified MSC_WT_ cells (Wilcoxon signed-rank test, *p* = 0.004) (Fig. 5). These results indicate that *CEBPB* plays a dual role in porcine mesenchymal stem cells, being essential not only for adipogenic differentiation but also for the regulation of cell proliferation.

## Discussion

A deeper understanding of the molecular mechanisms regulating genes essential for adipose tissue development is crucial not only for advancing obesity treatment strategies in humans but also for improving the nutritional and health-promoting qualities of meat in livestock through controlled fat deposition. Genome editing technologies, particularly CRISPR/Cas9, have been successfully applied in both human and rodent models, including adipose-derived mesenchymal stem cells (MSCs) (Khademi et al. 2024). Targeted genes in these models have been implicated in diverse biological processes such as adipogenesis, lipid metabolism, thermogenesis, and adipocyte browning (Kamble et al. 2020; Lopez-Yus et al. 2023; Karami et al. 2025). Pigs have been used as large-animal models for CRISPR/Cas9-mediated gene editing to study genes related to fat accumulation including *UCP1* (Tsagkaraki et al. 2021), *LGALS12* (Wu et al. 2024), and *SREBF1* (Aksoy et al. 2024).

In the present study, we selected the *CEBPB* gene, which encodes an early-acting transcription factor involved in adipogenesis, for CRISPR/Cas9-mediated disruption. Initially, we hypothesized that since *CEBPB* acts upstream of master regulators such as *PPARG* and *CEBPA*, its silencing would reduce—but not completely abolish—the adipogenic potential of MSCs. However, our results revealed that both homozygous and heterozygous deletions in the *CEBPB* promoter led to complete inhibition of adipogenesis in porcine MSCs. These findings are consistent with recent results from a study applying CRISPR interference (CRISPRi) in human preadipocytes, where *CEBPB* knockdown also blocked adipocyte differentiation. In that study, the authors observed reduced expression of adipogenic markers at days 0 and 8 of differentiation, including a > 60% reduction in *FABP4* expression at day 8 (Bielczyk-Maczyńska et al., 2023). The marked decrease in *CEBPB* transcript levels observed in both homozygous and heterozygous cells indicates that the reduction is primarily caused by the loss of promoter activity. However, our current data do not exclude potential compensatory mechanisms or indirect effects involving distal regulatory elements or chromatin architecture. Further analyses, including chromatin accessibility profiling and transcriptional activity assays, will be required to determine whether additional regulatory pathways contribute to the observed downregulation of *CEBPB*.

The complete inhibition of adipogenesis observed even in heterozygous *CEBPB* promoter-deletion clones suggests a strong dosage sensitivity of *CEBPB* expression during the early stages of adipocyte differentiation. This phenomenon may reflect haploinsufficiency, where a single functional allele is insufficient to maintain the threshold level of transcription factor activity required to initiate the adipogenic cascade. Although *CEBPB* is not typically classified as an allelic imbalance gene, as reported for other loci involved in adipogenesis and lipid metabolism in pigs (Stachowiak et al. 2018), its pronounced dosage dependence supports the notion that partial loss of promoter function can lead to a functionally haploinsufficient state. The data therefore indicate that adipogenesis in porcine mesenchymal stem cells is highly dependent on precise quantitative control of *CEBPB* expression.

In addition to its role in differentiation, our study demonstrates that *CEBPB* is also involved in regulating MSC proliferation. Cells with *CEBPB* promoter deletions exhibited significantly reduced expression of proliferation-associated genes (*CCND1*, *MCM2*, and *PCNA*), and those with homozygous deletions showed poor in vitro growth. While no prior studies have examined how CRISPR/Cas9-mediated *CEBPB* knockout affects MSC proliferation, data from CRISPRi experiments in preadipocytes suggest that *CEBPB* silencing alters early gene expression programs. Bielczyk-Maczyńska et al. (2023) found that the highest number of differentially expressed (DE) genes occurred at days 0 and 4 of differentiation following *CEBPB* knockdown. Although cell proliferation-related pathways were not explicitly enriched in their gene set enrichment analysis (GSEA), the analysis was limited to 10 hallmark gene sets. Moreover, studies in cancer biology have demonstrated that cells with *CEBPB* knockdown have impair cell proliferation (Wang et al. 2023; Tan et al. 2025), supporting our observation that this gene also influences proliferative capacity in porcine MSCs.

Given the pivotal role of *CEBPB* in initiating the adipogenic transcriptional cascade, targeting such an essential regulator with CRISPR/Cas9 results in complete blockade of differentiation and reduced proliferative capacity, making it difficult to investigate downstream regulatory mechanisms. Therefore, future genome editing experiments aimed at dissecting the transcriptional control of adipogenesis may benefit from focusing on genes with secondary or modulatory roles, whose disruption does not fully abrogate the differentiation process but rather alters specific pathways, such as lipid metabolism or energy homeostasis. For instance, Lopez-Yus et al. (2023) used CRISPR/Cas9 to knock down *SOCS3*, *SIK1*, and *DUSP1* in human adipose-derived MSCs. Although these genes were not essential for adipocyte formation, their silencing altered lipid accumulation and metabolic gene expression profiles, allowing a more nuanced interpretation of their functions without completely blocking adipogenesis. Similarly, in our previous study involving CRISPR/Cas9-mediated editing of the *SREBF1* locus (Aksoy et al. 2024), we demonstrated that deletion of the *SREBF1c* isoform affected the expression of genes acting upstream in the adipogenic cascade, including *CEBPB*. Notably, this disruption did not fully impair differentiation, which allowed for detailed functional analysis within the adipogenic network. These findings underscore the importance of gene selection in genome editing studies. Targeting genes that are upstream master regulators, may eliminate the possibility of studying adipogenesis altogether due to complete differentiation arrest. In contrast, focusing on regulatory or downstream effectors—such as transcriptional modulators, co-factors, or isoforms—may enable more informative experiments that preserve cell viability and still providing mechanistic insight into adipose biology.

In conclusion, we demonstrated that CRISPR/Cas9-mediated deletion of the *CEBPB* promoter region resulted in a complete inhibition of adipogenic differentiation and a significant reduction in the proliferative potential of porcine mesenchymal stem cells. These findings indicate that *CEBPB* is essential not only for initiating adipogenesis but also for maintaining MSC proliferation. Therefore, in future experiments aimed at generating gene-edited pigs with modified adiposity, it is advisable to select target genes that do not completely impair both proliferation and differentiation processes.

## Supplementary Information

Below is the link to the electronic supplementary material.

Supplementary Material 1
