# Supplementary material for: The role of the CEBPB gene in porcine adipogenesis: a study using CRISPR/Cas9-edited mesenchymal stem cells
Source: J Appl Genet. 2025 Nov 26;67(2):477–86. doi: 10.1007/s13353-025-01030-x (PMC13079538; doi:10.1007/s13353-025-01030-x)
Supplement: Supplementary file 1 — Supplementary Material 1 [file 13353_2025_1030_MOESM1_ESM.pdf]

Supplementary materials

The Role of the *CEBPB* Gene in Porcine Adipogenesis: A Study Using CRISPR/Cas9-Edited Mesenchymal Stem Cells

Mehmet Onur Aksoy, Jedrzej Rozynek, Monika Stachowiak, Izabela Szczerbal*

Table S1 gRNAs/primers directed against the 5'flanking region of *CEBPB* gene.

	**gRNA**	**Name**	**Sequence**
CEBPB	gRNA5	CEBPb_g5_F	caccgCCGCGGCCGCGTTTAGGGT
		CEBPb_g5_R	aaacACCCTAAACGCGGCCGCGGc
	gRNA7	CEBPb_g7_F	caccgCGCGTTCATGCAACGCCTGG
		CEBPb_g7_R	aaacCCAGGCGTTGCATGAACGCGc

Table S2 Sequences of primers designed for genotyping single MSC colonies.

**Primer name**	**Sequence**	**Annealing temperature**
**CEBPb_geno_F**	GGGCTACCTCTCCAATAGCC	60°C
**CEBPb_geno_R**	AAGCAGTCCGCCTCGTAGTA	

Table S3 List of primer pairs used in RT-qPCR.

**Gene**	Primer sequence	Product size (bp)	Annealing temperature (°C)	Genbank accession number
RPL27*	F: 5` GCAAAGCGGTCATCGTAAA<br>R: 5` CTTGTGGGCATGAGGTGAT	190	60	NM_001097479.1
PPARG	F: 5` GCATCAGCTCTGTGGACCTG<br>R: 5` GATCAGCTCTCGGGAATGGG	132	60	XM_005669783
CEBPA	F: 5` CGTGAGCGCAACAACATCG<br>R: 5` CTCAGTTGTTCCACCCGCTT	131	60	NC_010448
FABP4	F: 5` TTCAAATTGGGCCAGGAAT<br>R: 5` ATTCTGGTAGCCGTGACACC	191	60	NM_001002817
CEBPB	F: 5` TACTACGAGGCGGACTGCTT<br>R: 5` TCCAGGTATGGGCTGAAGTC	152	60	NM_001199889.1
GATA2	F: 5` CTCCAGCTTCACCCCTAAG<br>R: 5` CCCGTTCATCTTGTGGTACAG	157	60	XM_021068525.1
CCND1	F: 5` GCCGAGAAGTTGTGCATCTA	140	60	NC_010444

	R: 5` TTGGAGAGGAAGTGCTCGAT			
MCM2	F: 5` AGCATCGCTCCTTCCATCTA<br>R: 5` CACAGGAGCACGTTGATGTC	128	60	NC_010455
PCNA	F: 5` GATTTAGATGTTGAGCAACTTGG<br>R: 5` GCACAGGAAATTACAACAGCA	131	60	NC_010459

* *reference gene*

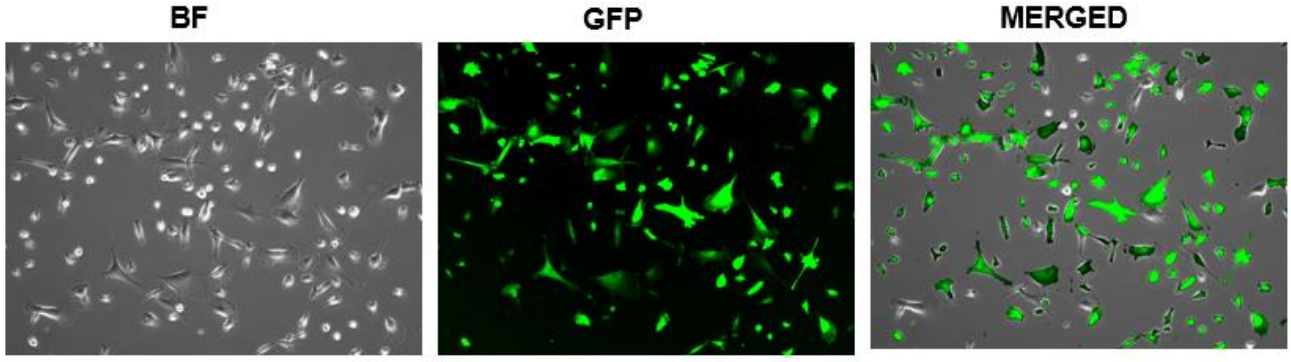

Fig. S1. Evaluation of transfection efficiency in MSCs using the pmaxGFP control plasmid (Lonza, Basel, Switzerland). Fluorescence microscopy images obtained 24 h after transfection show GFP expression in approximately 60% of cells, as determined by counting GFP-positive cells in five randomly selected microscopic fields. BF: bright field; GFP: green fluorescent protein channel; MERGE: fusion.

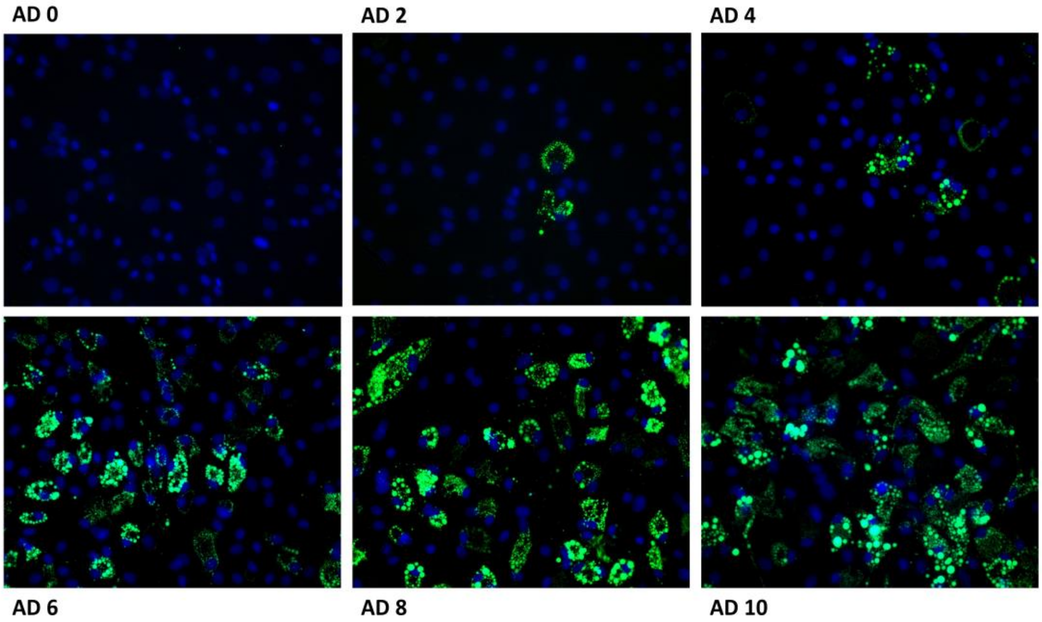

Fig. S2 BODIPY staining of wild-type mesenchymal stem cells (MSC_{WT}) showing progressive accumulation of lipid droplets during adipogenic differentiation (AD) on days 0, 2, 4, 6, 8, and 10 (AD0–AD10). In contrast, modified cells (MSC_{DEL}) failed to accumulate lipid droplets, indicating a lack of adipogenic potential following *CEBPB* promoter deletion.
